# Time-Resolved Förster Resonance Energy Transfer Nanoassay Based on CdTe Quantum Dots for Sensitive Detection of Prostate Cancer Antigen 3

**DOI:** 10.1021/acsanm.5c02176

**Published:** 2025-07-08

**Authors:** Catarina S. M. Martins, Anne Nsubuga, Nour Fayad, Ruifang Su, Thibault Gallavardin, Ihsan Çaha, Niko Hildebrandt, Francis Leonard Deepak, João A. V. Prior

**Affiliations:** † LAQV, REQUIMTE, Laboratory of Applied Chemistry, Department of Chemical Sciences, 131671 Faculty of Pharmacy of the University of Porto, Porto 4050-313, Portugal; ‡ 246702 International Iberian Nanotechnology Laboratory, Braga 4715-330, Portugal; § Laboratoire COBRA, 27040 Université de Rouen Normandie, CNRS, INSA, Rouen 76821, France; ∥ Laboratory of Biomaging and Pathologies, UMR 7021 CNRS, 27083 University of Strasbourg, Strasbourg 67000, France; ⊥ 3710 McMaster University, Department of Engineering Physics, Hamilton L8S 4L7, Canada

**Keywords:** diagnostics, lncRNA, PCA3, quantum dots, terbium, TR-FRET

## Abstract

Prostate Cancer Antigen 3 (PCA3) is a long noncoding RNA highly expressed in prostate cancer cells, making it a promising biomarker for noninvasive prostate cancer diagnosis. Simple and rapid detection using nanoprobes can potentially overcome the limitations of traditional diagnostic techniques. Here, we designed, characterized, and applied a DNA-strand displacement assay based on Förster Resonance Energy Transfer (FRET) between terbium (Tb) ions and semiconductor quantum dots (QDs) as a proof-of-concept for sensitive and specific mix-and-measure quantification of a synthetic DNA analogue of PCA3. The assay utilized QDs synthesized through an aqueous one-pot method. The time-resolved (TR) FRET assays achieved a detection limit of 0.65 nmol L^–1^ by using a *SPARK* benchtop fluorescence plate reader and 0.32 nmol L^–1^ by using a *KRYPTOR Compact PLUS* clinical plate reader. Despite slightly decreased performance, the TR-FRET nanoassay demonstrated reliable quantification of nanomolar PCA3 concentrations also in samples containing up to 50% of serum. These findings underscore the potential of Tb-to-QD FRET assays for rapid clinical prostate cancer diagnostics, offering a promising tool for the early detection of PCA3 in a noninvasive manner.

## Graphical abstract

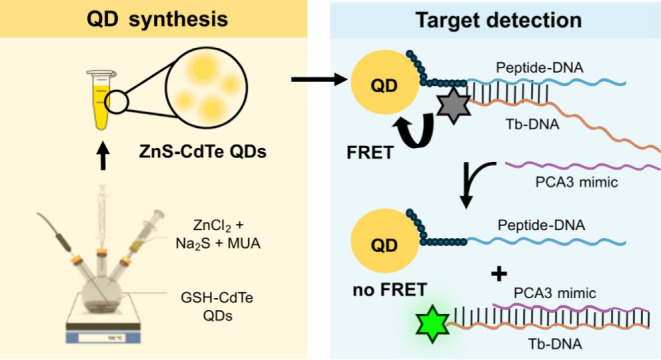

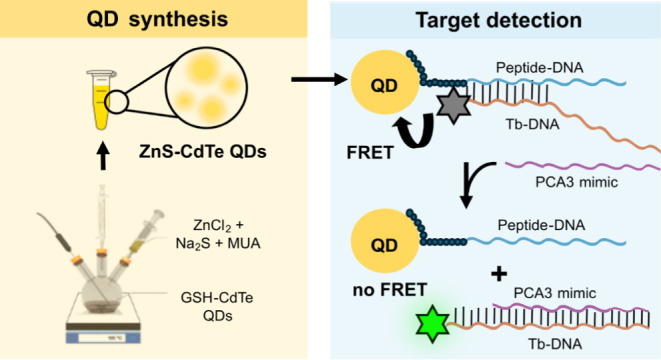

## 1 Introduction

The most recent data from GLOBOCAN 2022 ^1^ show prostate cancer (PCa) as the second most frequently diagnosed cancer in men globally, being the third cause of cancer-related death, and in 2020, across Europe, it accounted for 335 514 new cases diagnosed in men.^2^ The incidence of PCa worldwide reflects the variations in cancer management and treatment, being higher in developed regions due to widespread prostate-specific antigen (PSA) testing, while mortality rates are elevated in areas with limited access to early diagnosis and treatment.^3^ Although often asymptomatic in early stages, leading to challenges in detection, PSA testing has increased diagnosis rates, raising concerns about overdiagnosis and overtreatment, due to its low specificity.^4^ It is known that PSA alone is not a cancer-specific biomarker, and high levels of PSA can also occur in benign prostatic hyperplasia, prostatitis, due to normal aging, and other nonmalignant conditions.^5,6^ Besides the PSA test, digital rectal examination, magnetic resonance imaging, ultrasound-based techniques, and blood and urine biomarkers are other primary diagnostic tools that are present in the European guidelines. Depending on the primary diagnostic results, a more invasive approach, such as a biopsy, can be applied to make sure of the diagnosis of PCa.^7^ However, challenges persist in accurately identifying patients who truly need biopsies to prevent unnecessary procedures and in treating the aggressive forms of the disease.

Prostate Cancer Antigen 3 (PCA3) is a long noncoding RNA (lncRNA) that is overexpressed in PCa cells and can be detected, more specifically, in urine samples collected after a digital rectal examination. It can be used as a biomarker for PCa diagnosis as well as for patient follow-up, prognosis prediction, and targeted therapy. Notably, PCA3 is uniquely expressed in prostate cancer (PCa) cells and remains unaffected by the prostate size or other benign conditions. When used alongside the PSA test, it has been studied as a more precise diagnostic tool.^8,9^ Several analytical methods for monitoring PCA3 in urine samples have been proposed. These include methods based on colorimetric detection,^10^ surface-enhanced Raman scattering,^11,12^ and electrochemical impedance spectroscopy.^13,14^ For instance, Rodrigues, V., et al.^15^ used commercial carbon electrodes, coated with alternating layers of gold nanoparticles and chondroitin sulfate, and through electrochemical impedance spectroscopy, the authors accomplished the detection of PCA3 mimic DNA, with a limit of detection (LOD) of 83 pmol L^–1^. On the other hand, Fu, X. et al.^11^ developed a surface-enhanced Raman scattering-based competitive lateral flow assay capable of detecting PCA3 mimic DNA at the femtomolar level, with a LOD of 3 fmol L^–1^. Additionally, Yu, J. et al.^12^ applied surface-enhanced Raman scattering to detect PCA3 mimic DNA. In their approach, the target PCA3 sequence was hybridized between two probes: magnetic beads and hollow gold nanospheres, each functionalized with antisense oligonucleotides, ASO683 and ASO735, respectively. This detection method employed a sandwich assay format, where the Raman signal intensity was monitored, achieving an LOD of 2.7 fmol L^–1^.

Although different methodologies exhibit varying levels of limit of detection and sensitivity, it is important to also consider the time and cost involved in sensor production as well as the need for specialized detection equipment not commonly available in clinical laboratories. Therefore, fluorescence biosensing is also a promising technique for measuring PCA3 due to its rapid response and high sensitivity. Examples of fluorescence-based methods include the use of functionalized titanium carbide MXenes,^16^ upconversion nanoparticles,^17^ or Ag_2_S quantum dots (QDs). For example, Jia et al.^18^ designed a biosensor to detect PCA3 based on the metal-enhanced fluorescence effect, and to the best of our knowledge, this is the only study that reported the detection of PCA3 using the native fluorescence of quantum dots. Jia et al.^18^ presented a near-infrared fluorescent quantitative analysis method for the detection of PCA3 based on an Au nanorod/Ag_2_S quantum dot satellite structure. The sensor was composed of DNA-functionalized gold nanorods and Ag_2_S quantum dots, wherein in the presence of the target, a satellite structure is formed and an optimal distance between both probes is made, leading to the metal-enhanced fluorescence effect. The proposed sensing scheme exhibited a linear correlation within the 5–500 pmol L^–1^ range and a detection limit of 1.42 pmol L^–1^. In this work, the problem of autofluorescence background from complex biosystems was overcome by using fluorescent probes with NIR emissions.

Thus, the use of QDs in the detection of PCA3 from real samples can be an alternative for other noninvasive methods for prostate cancer diagnosis, reducing the need of more invasive procedures like tissue biopsies. Quantum dots are nanometer-sized semiconductor particles with unique optical and electronic properties, making them highly valuable in biomedical applications, including cancer diagnosis.^19^ They can emit light when excited by a light source, and their size and composition can be precisely controlled, allowing them to emit at specific wavelengths. The tunable fluorescence, ability to be functionalized with molecular recognition elements, along with their high brightness and stability make QDs ideal for imaging and detecting biomolecules.^19^

One possibility of rapid and specific diagnostic testing based on fluorescence is the application of Förster Resonance Energy Transfer (FRET). Due to their homogeneous (mix-and-measure without separation and washing steps) format, FRET assays have found broad application in molecular diagnostics.^20^ In particular, time-gated (TG)- or time-resolved (TR)-FRET assays using lanthanide-based donors can significantly improve signal-to-background ratios because the autofluorescence of biological samples can be efficiently suppressed.^21^ Combining the advantages of TR-FRET with those of QDs can result in important benefits concerning multiplexed detection with high sensitivity and very photostable photoluminescence (PL).^22–24^ Considering that terbium (Tb)-to-QD FRET has been successfully applied for the multiplexed quantification of DNA,^25^ microRNA,^26^ or protein cancer biomarkers,^27^ it holds a great potential for the development of PCA3 diagnostic assays.

In order to establish a proof-of-concept for quantifying circulating PCA3 lncRNA via Tb-to-QD TR-FRET, we developed a DNA-strand displacement assay in which hybridized Tb-DNA and QD-DNA probes are displaced by the hybridization of a DNA analogue of PCA3 to Tb-DNA (Figure 1). This occurs because the analyte PCA3 has a stronger binding affinity to Tb-DNA due to its 30-base complementary sequence, whereas the QD-DNA has only 11 bases that are complementary to the Tb-DNA, resulting in a weaker interaction. This strand displacement interrupts Tb-to-QD FRET and recovers the Tb PL (previously quenched via Tb-to-QD FRET), such that the PCA3-DNA concentration is proportional to the Tb PL intensity (Tb PL turn-on sensor). The rapid mix-and-measure assay did not require any separation or washing steps, and the Tb-DNA and QD-DNA probes were produced via simple bioconjugation of the *N*-hydroxysuccinimide (NHS)-functionalized Tb complex Lumi4-Tb to amino-functionalized DNA and metal-affinity-mediated self-assembly of CdTe/ZnS core/shell QDs to hexa-histidine (His_6_)-appended DNA.^28^ Notably, the QDs were synthesized via microwave irradiation in open-air conditions, through a rapid one-pot aqueous synthesis at low temperature.^29^

**Figure 1:**
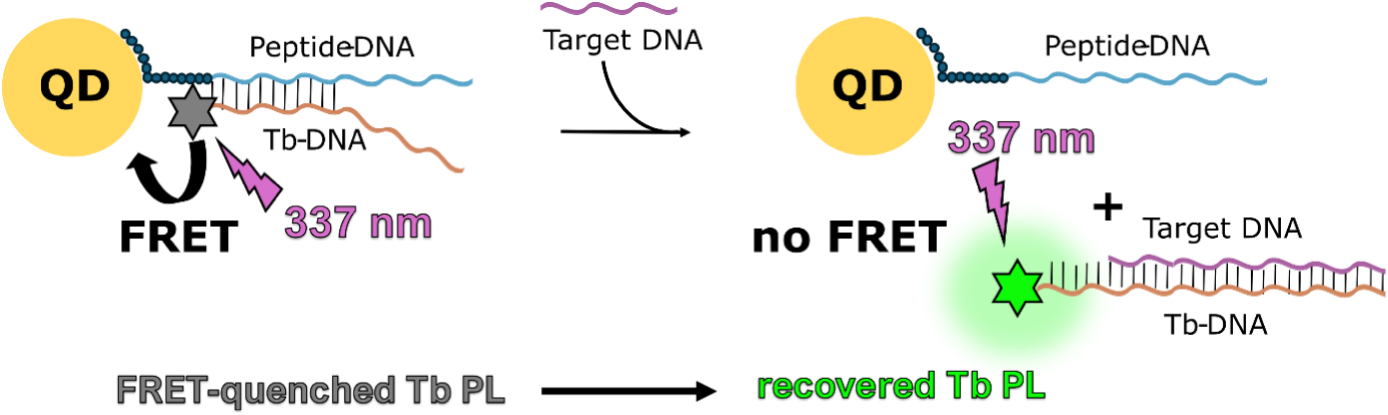
Schematic presentation of the Tb-to-QD FRET DNA-displacement assay. QD-DNA and Tb-DNA are partially complementary, forming an 11 nucleotide (nt) dsDNA that enables Tb-to-QD FRET, which results in Tb PL quenching (left). The PCA3 target DNA provides a 30 nt complementarity with Tb-DNA, resulting in the displacement of Tb-DNA from QD-DNA to the target DNA and a concomitant recovery of Tb PL due to the interruption of Tb-to-QD FRET (right). Thus, the Tb PL intensity is proportional to the PCA3 target concentration. The absorption and emission spectra of Tb and QD are shown in Figure 2.

## 2 Materials and Methods

### 2.1 Reagents

All of the following chemicals were used without any treatment process or further purification. Cadmium chloride (CdCl_2_, 99.99%, Sigma-Aldrich, USA), citrate (Na_3_C_6_H_5_O_7_·2H_2_O, trisodium citrate dihydrate, Merck, Germany), sodium tellurite (Na_2_TeO_3_, 99%, ≈100 mesh, Sigma-Aldrich, USA), l-reduced glutathione (GSH ≥ 98%, Sigma-Aldrich, USA), sodium borohydride (NaBH_4_, ReagentPlus, 99%, Sigma-Aldrich, USA), sodium hydroxide (NaOH, VWR Chemicals, Belgium), hydrochloric acid (HCl, Sigma-Aldrich, USA), zinc chloride (ZnCl_2_, Acros Organics, Belgium), sodium sulfide hydrate (Na_2_S·H_2_O, 60–62%, Acros Organics, Belgium), and 11-mercaptoundecanoic acid (MUA, 95%, Sigma-Aldrich, USA). All solutions were prepared with water purified from a Milli-Q system (conductivity ≤ 0.1 μS cm^–1^), with the exception of MUA, that was dissolved in *N*,*N*-dimethylformamide (DMF, 99.8%, Sigma-Aldrich). Lumi4-Tb complexes functionalized with NHS were provided by Lumiphore Inc. Trizma hydrochloride, sodium carbonate (Na_2_CO_3_), sodium bicarbonate (NaHCO_3_), sodium chloride (NaCl), bovine serum albumin (BSA), and fetal bovine serum (FBS) were purchased from Sigma-Aldrich, USA. Custom oligonucleotides (ssDNA) were purchased from Eurogentec, Belgium. Peptide DNA sequence hexa-histidine (His_6_) tag (His_6_-DNA) was purchased from BioSynthesis, USA. The purification of Tb-DNA was performed using Zeba Spin Desalting Columns (7 kDa MWCO). All oligonucleotides’ sequences used, as well as peptide-DNA, are described in Table 1.

**Table 1:** Summary of All Sequences and Modifications of DNA Oligonucleotides Used for FRET Experiments^a^

Sequence Name	Internal name	Sequence (5′ to 3′)	nt	EE^b^ (L mol^–1^ cm^–1^)	Modification
Peptide DNA	-	**AGTCTAGTGC**GACACGACA	19	191 600	5′-*N*-H6SLGAAAGSGC-SMCC-amino
Tb-DNA	NBP770	GTATGATTTGCCAAAATTCTAAA**GCGCACTAGACT**	35	344 000	3′-C_6_ amino
Target PCA3	NBP768	AGTGCGCTTTAGAATTTTGGCAAATCATAC	30	293 200	-
Nontarget DNA	NBP767	GACAAATACCTAATGCATGTGGGACTTAAA	30	306 800	-
Nontarget DNA	NBP353	CAACGGAATCCCAAAAGCAGCTG	23	227 800	-

a Internal names correspond to the laboratory nomenclature for these oligonucleotides.

b EE: molar extinction coefficient at 260 nm.

### 2.2 Instrumentation

The synthesis of GSH-capped CdTe QDs was carried out using a microwave synthesizer operated by Synergy software (CEM Discover SP, Matthews, NC, USA), following an adapted procedure based on Martins et al.^29^ The reactions were carried out in 35 mL borosilicate glass vessels with controlled reaction temperature (30–300 °C), pressure (0–200 psi), power (0–300 W), and stirring. The ZnS shell synthesis was performed under reflux, using a round-bottom, three-neck flask and a heater plate with stirring and temperature control. The solutions were injected using a syringe pump (Pump 11 Elite, Harvard Apparatus, USA). Absorption spectra of QDs and Tb were recorded with a Cary 5000 Scan ultraviolet-visible spectrophotometer (Varian) in the 200–800 nm range. Emission spectra of Tb and QDs were recorded in HEPES medium (100 mM, pH 7.4) using a Fluorolog-3 (Horiba Instruments) with an excitation wavelength of 337 nm. For morphological, structural, and chemical analysis, transmission electron microscopy (TEM) was performed using a JEOL JEM 2100 model TEM and an FEI Titan ChemiSTEM equipped with a Cs probe corrector and a SuperX EDX system, operated at 200 kV. Sample preparation involved placing droplets of QD suspension onto a 200-mesh copper grid (Ted Pella Inc., CA, USA) and allowing them to dry. Time-resolved photoluminescence (TR PL) intensity measurements were obtained using a multimode fluorescence plate reader *SPARK* (Tecan, Switzerland) with an excitation wavelength of 337 nm (20 nm bandwidth), an emission range of 450–700 nm, a lag time of 100 μs, an integration time of 2000 μs, and a gain of 150. Additionally, measurements were performed using the *KRYPTOR Compact PLUS* clinical fluorescence plate reader (Thermo Fisher Scientific, Germany) within a time window from 0.1 to 0.9 ms postpulsed excitation at 337.1 nm, utilizing an integrated nitrogen laser operating at 20 Hz with 100 pulses. A bandpass filter (Semrock, NY, USA) at 494 ± 10 nm was used for detection of Tb PL. Data analysis and graph plotting were carried out using Origin 9 software (OriginLab, Massachusetts, USA). Image data were processed and analyzed using ImageJ software (Maryland, USA).

### 2.3 Reagent Setup

The oligonucleotide suspensions were prepared by resuspending custom oligonucleotides (ssDNAs) in nuclease-free water to a concentration of 100 μM and stored at −20 °C. For FRET assays, the following buffers were prepared: a Hybridization Buffer (HB) containing 20 mM Tris, 500 mM NaCl, 2 mM MgCl_2_, and 0.1% BSA at pH 8.0 (25 °C); a 100 mM HEPES buffer at pH 7.4 (25 °C); and a 100 mM carbonate buffer at pH 9.0 (25 °C).

### 2.4 Synthesis of Core–Shell QDs

The GSH-capped CdTe QDs were synthesized by using a one-step synthetic route assisted by microwave irradiation. Briefly, 19 mL of water was added to a beaker and maintained under stirring. Then, 2.5 mL of CdCl_2_ stock solution at 0.1 mol L^–1^ was introduced, followed by the addition of the same amount of citrate stock solution at 0.1 mol L^–1^. Subsequently, 76.80 mg of L-reduced GSH was added. Next, 1.0 mL of Na_2_TeO_3_ (0.05 mol L^–1^) solution was added to the beaker, and the resulting mixture was transferred to a new beaker containing preweighed 30.26 mg of NaBH_4_. The pH was adjusted to 9.8 using either 1 or 0.5 mol L^–1^ NaOH. Finally, the mixture was poured into a reaction vessel and subjected to microwave irradiation, for 30 min at 107 °C, using a CEM Discover SP microwave synthesizer (200 PSI and 200 W). The produced GSH-QDs were thoroughly characterized to assess their properties, and their concentration was accurately determined.

For the synthesis of the ZnS shell, an easy one-pot approach was applied, adapted from Saikia et al.^30^ 3 mL of CdTe QDs (37 μmol L^–1^) were transferred to a round-bottom three-neck flask and then heated at 100 °C with reflux. After the temperature was reached, aqueous solutions of ZnCl_2_ (5.11 × 10^–5^ mol L^–1^, 1 mL) and Na_2_S (5.11 × 10^–5^ mol L^–1^, 1 mL) were injected at the same time, with an injection rate of 0.2 mL·min^–1^. After that, 11-mercaptoundecanoic acid (MUA) (3.06 × 10^–4^ mol L^–1^, 0.5 mL) was also injected with the same injection rate. The mixture was allowed to reflux for 75 min. The resultant core–shell QDs were kept at 4 °C for further use.

### 2.5 Characterization of Core–Shell QDs

The optical characterization of QDs was performed by UV–vis and fluorescence spectroscopy, and their size (*D*) in nm was estimated through eq 1 and also confirmed by TEM. The extinction coefficient (ε, L mol^–1^ cm^–1^) was also estimated by using eq 2.^31^

1 $$D=\left(9.8127\times 10^{-7}\right)\lambda^{3}-\left(1.7147\times 10^{-3}\right)\lambda^{2}+\left(1.0064\right)\lambda -\left(194.84\right)$$

2 $$\varepsilon =3450\times \Delta E\times D^{2.4}$$

3 A=ε×l×C

Herein, λ (nm) is the wavelength of the first excitonic absorption peak of the corresponding sample, and Δ*E* represents the transition energy associated with the first absorption peak, measured in eV. To determine the concentration (*C*), in mol L^–1^, of the nanocrystals, eq 3 was used, in which *A* is the absorbance at the peak position of the first exciton absorption maximum, and *I* is the path length (cm) of the cuvette used for recording the absorption spectrum. In this study, *I* was fixed at 0.3 cm, and ε was previously calculated (eq 2).

### 2.6 Tb-DNA Conjugation

Tb-DNA conjugation was performed as described in the study.^32^ In summary, 10 μL of 100 μmol L^–1^ amino-functionalized oligonucleotide was mixed with 2.1 μL of 8 mmol L^–1^ Lumi4-Tb-NHS (in anhydrous DMF) and 7.9 μL of 100 mmol L^–1^ carbonate buffer at pH 9, at 25 °C. In this mixture, Tb-NHS was in 16-fold molar excess relative to the oligonucleotide. The solution was vortexed and incubated overnight at 4 °C. The Tb-DNA conjugate was purified three times from Lumi4-Tb and eventual impurities by using Zeba Spin Desalting Columns (7 kDa MWCO) according to the manufacturer’s desalting protocol. The concentration of the purified conjugate and the labeling ratio were determined from absorbance measurements at 260 nm (oligo, ε = 344 000 L mol^–1^ cm^–1^) and 340 nm (terbium, ε = 26 000 L mol^–1^ cm^–1^) using a Cary 5000 Scan ultraviolet-visible spectrophotometer. According to eq 3, the concentration of Tb-DNA is 5.96 μmol L^–1^ and has a Tb-to-oligo ratio of 1:2.

### 2.7 FRET Parameters

The overlap integral (*J*) from 450 to 700 nm, Förster distance (*R*_0_), and FRET efficiency (*E*_FRET_) were calculated using the following equations:^33^

4 $$J=\int_{450}^{700}I_{D}\left(\lambda \right)\varepsilon_{A}\left(\lambda \right)\lambda^{4}d\lambda$$

5 $$R_{0}={\left(0.02108{\;\times \;\kappa }^{2}{\;\times \phi }_{D}{\;\times n}^{-4}\;\times J\right)}^{1/6}\mathrm{nm}$$

6 $$E_{\mathrm{FRET}}=\frac{1}{1+{\left(\frac{r}{R_{0}}\right)}^{6}}=1-\frac{I\left(\mathrm{Tb}/\mathrm{QD}\right)}{I\left(\mathrm{Tb}\right)}$$

Here, *I*_D_(λ) represents the emission intensity derived from the emission spectrum of the Tb donor, which has been normalized to an area of unity. ε_A_(λ) corresponds to the molar absorptivity of the acceptor (ZnS CdTe QDs). The spectral overlap is represented in Figure 2.

**Figure 2:**
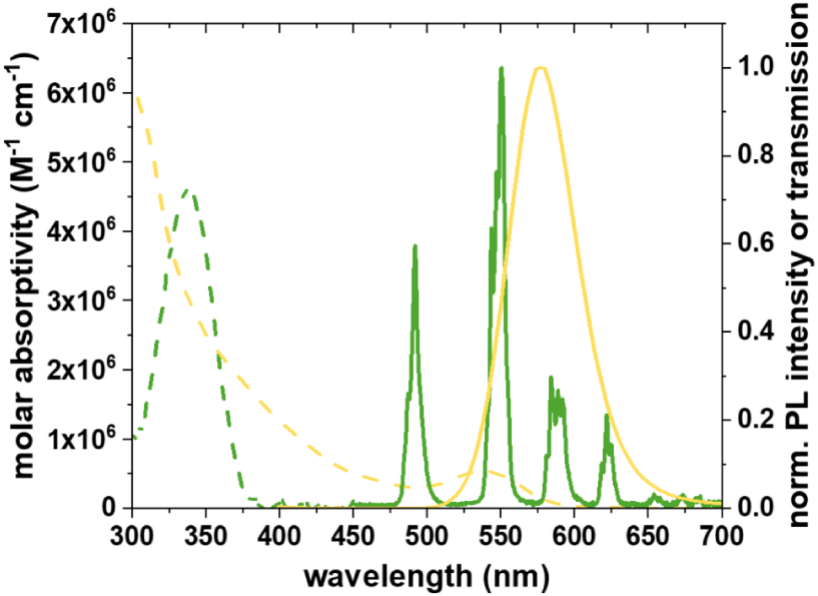
PL emission spectral overlap of donor emission (Tb, green line) and acceptor absorption (ZnS CdTe QDs, yellow line). QD absorption spectra (yellow dotted line) showed good spectral overlap with the Tb PL. Excitation at 337 nm was close to the maximum absorption of Tb (green dotted lines).

The orientation factor (κ^2^) was assumed to be 2/3, reflecting the random orientation of the donor and acceptor during the FRET process. This assumption is justified by the dynamic averaging effect, supported by the extended PL lifetime of the Tb donors and their unpolarized emission, indicating rapid isotropic rotation.^33^ The refractive index of the medium was set to 1.35, consistent with the properties of the aqueous solution. The PL quantum yield of the central Tb ion was 0.81 ± 0.05.^34^ *r* is the distance between the donor and acceptor, in this case, the Tb-to-QD distance. *I*(Tb/QD) and *I*(Tb) represent the Tb PL intensities in the presence and the absence of the QD acceptor, respectively.

The Lumi4-Tb donor used in this work has been previously applied for various TR-FRET assays with QD or dye acceptors.^21,35^ The long Lumi4-Tb photoluminescence lifetime of ca. 2.7 ± 0.2 ms is essential for enabling time-gated detection and supports efficient energy transfer when paired with quantum dot acceptors, as assumed in our calculations using a Förster radius of 6.6 ± 0.4 nm.

### 2.8 FRET Assays

#### 2.8.1 Assay Protocol

All assays for the detection and quantification of PCA3-DNA were conducted under the same experimental conditions and measurement parameters except when noted otherwise. The samples prepared in each assay were incubated using the same protocol: 30 min at 37 °C, followed by a 2 h incubation at room temperature. Also, the data from all experiments were assessed based on the relative TR PL intensities of Tb recorded at 494 nm (one of the major peaks of the Tb emission, Figure 2). The relative intensity was calculated as the ratio of TR PL intensity at a specific target concentration to TR PL intensity in the absence of the target (eq 7):

7 $$\mathrm{Relative}\;\mathrm{PL}\;\mathrm{Intensity}=\frac{I_{\mathrm{Tb}\left(c=x\right)}}{I_{\mathrm{Tb}\left(c=0\right)}}$$

#### 2.8.2 Optimization of the DNA Probes

To optimize the conditions for preparing the DNA probes, the effects of His_6_-DNA and Tb-DNA concentrations on the PL signal of the probes were assessed. For the study of His_6_-DNA concentration, 50 μL of ZnS-CdTe QDs (3.33 nmol L^–1^) and 50 μL of Tb-DNA (6.67 nmol L^–1^) were combined with 50 μL of His_6_-DNA at varying concentrations (0–12.0 nmol L^–1^). The incubation procedure was described in the “Assay Protocol” section. In the Tb-DNA experiment, 50 μL of ZnS-CdTe QDs (3.33 nmol L^–1^) and 50 μL of His_6_-DNA (10.0 nmol L^–1^) were mixed with 50 μL of Tb-DNA at different concentrations (0–12.0 nmol L^–1^). These mixtures also underwent an incubation step, as described in the “Assay Protocol” section. All the above concentrations are those already in the total assay volume of 150 μL. For all experiments, each concentration of His_6_-DNA and Tb-DNA was tested in triplicate (*n* = 3).

#### 2.8.3 Detection of Target PCA3

A mixture of 100 μL of the probe (containing ZnS QDs at 3.33 nmol L^–1^, Tb-DNA at 6.67 nmol L^–1^, and His_6_-DNA at 10.0 nmol L^–1^) in HB was combined with 50 μL of the target, a short ssDNA sequence that mimics the PCA3 lncRNA, hereafter designated as target PCA3 (see Table 1). The target concentration ranged from 0 (sample blank) to 10.0 nmol L^–1^, yielding the stated final concentrations in the total assay volume of 150 μL. Then, the samples were incubated as described in the “Assay Protocol” section. Each concentration was prepared in triplicate (*n* = 3), except for the sample blank (*n* = 10). Limits of detection (LODs) were determined as the concentrations on the assay calibration curves that produced a signal exceeding three standard deviations above the zero control.

#### 2.8.4 PCA3 Sensitivity and Specificity Assay

The mixture of 100 μL of the DNA probes (ZnS QDs at 3.33 nmol L^–1^, Tb-DNA at 6.67 nmol L^–1^, His_6_-DNA at 10.0 nmol L^–1^) in HB was mixed with 50 μL of target or nontarget DNA, at concentrations from 0 to 1.5 nmol L^–1^, and incubated as described in the “Assay Protocol” section. Each concentration was prepared in triplicate (*n* = 3).

#### 2.8.5 Spike-In Test with Fetal Bovine Serum

The responsiveness of the developed nanosensor in more realistic samples, specifically serum samples, was tested by monitoring the target in various concentrations of fetal bovine serum. The control calibration curve was performed in buffer (0% serum): a mixture of 100 μL of the probe (ZnS QDs at 1.67 nmol L^–1^, Tb-DNA at 3.33 nmol L^–1^, His_6_-DNA at 5.00 nmol L^–1^) in HB, and 50 μL of target DNA in various concentrations from 0 to 10.0 nmol L^–1^. For the following calibration curves, the probe was mixed with 5%, 10%, 20%, and 50% of FBS for the total volume. All experiments underwent an incubation step, as described in the “Assay Protocol” section.

## 3 Results and Discussion

### 3.1 Preparation of the Detection Probes

The TR-FRET nanosensor (Figure 1) was composed of two different DNA hybridization probes. The first one consisted of His_6_-peptide-appended ssDNA that self-assembled to the Zn-rich surface of CdTe/ZnS core/shell QDs (abbreviated as QD-DNA throughout the manuscript). The second one consisted of a complementary ssDNA that was labeled on its 3′ end with a Lumi4-Tb complex (abbreviated as Tb-DNA throughout the manuscript). The Tb-QD donor–acceptor FRET pair was formed via hybridization of QD-DNA with Tb-DNA, resulting in quenched Tb PL. The spectral overlap of Tb emission and QD absorption (Figure 2) was used to calculate the Förster distance (eq 5) as *R*_0_ = 6.6 ± 0.4 nm. When considering a QD diameter of approximately 3 nm and that the Tb donor binds very close to the QD (cf., Figure 1 left), this *R*_0_ value should be sufficient for efficient Tb-to-QD FRET upon QD-DNA/Tb-DNA hybridization.

#### 3.1.1 Synthesis and Characterization of Core–Shell QDs

GSH-capped CdTe QDs were prepared via a rapid aqueous-phase low-temperature microwave synthesis, followed by an additional step to form a ZnS shell around the QD core. The ZnS shell did not only provide efficient surface passivation for improved photophysical properties, enhanced biocompatibility, and reduced toxicity^36^ but also added a Zn-rich nanosurface for the self-assembly of the His_6_-tagged peptide DNA. This allows for simple and rapid QD-DNA bioconjugation without the necessity of separation steps and can be performed right before the assay experiments through metal-affinity coordination. A one-pot synthesis approach was applied to grow the shell around the QD surface. The CdTe/ZnS QDs showed a first exciton absorption peak at around 530 nm and an emission maximum at around 575 nm (Figure 2). The relative photoluminescence quantum yield of the TGA-capped CdTe QDs (prior to ZnS shell growth) was determined to be approximately 53.2%, indicating efficient emission properties before shell modification. The average particle size (Figure 3A) was determined by TEM measurements, exhibiting a spherical shape (Figure 3B) and a diameter of 3.3 ± 0.5 nm, which was in agreement with the nanocrystal size according to eq 1 (3.1 nm) and a concentration of 15 μmol L^–1^ (eq 3). A representative selected area electron diffraction (SAED) pattern is depicted in Figure 3D, showing five distinct diffraction rings that correspond to the (220), (422), (440), (533), and (822) planes of the F4̅3m space group cubic crystal structure of CdTe/ZnS QDs. The Fast Fourier Transform (FFT) pattern (Figure 3E) obtained from a single particle, as shown in Figure 3C, clearly shows the reflections corresponding to the CdTe QD covered by the ZnS shell. The high-resolution TEM (HRTEM) image in Figure 3C shows the highlighted (111) plane of the CdTe QD with the (220) plane of the ZnS shell. This shell formation was also confirmed by the EDX spectrum (Figure 3F) where we can see Zn, S, Cd, and Te elements.

**Figure 3:**
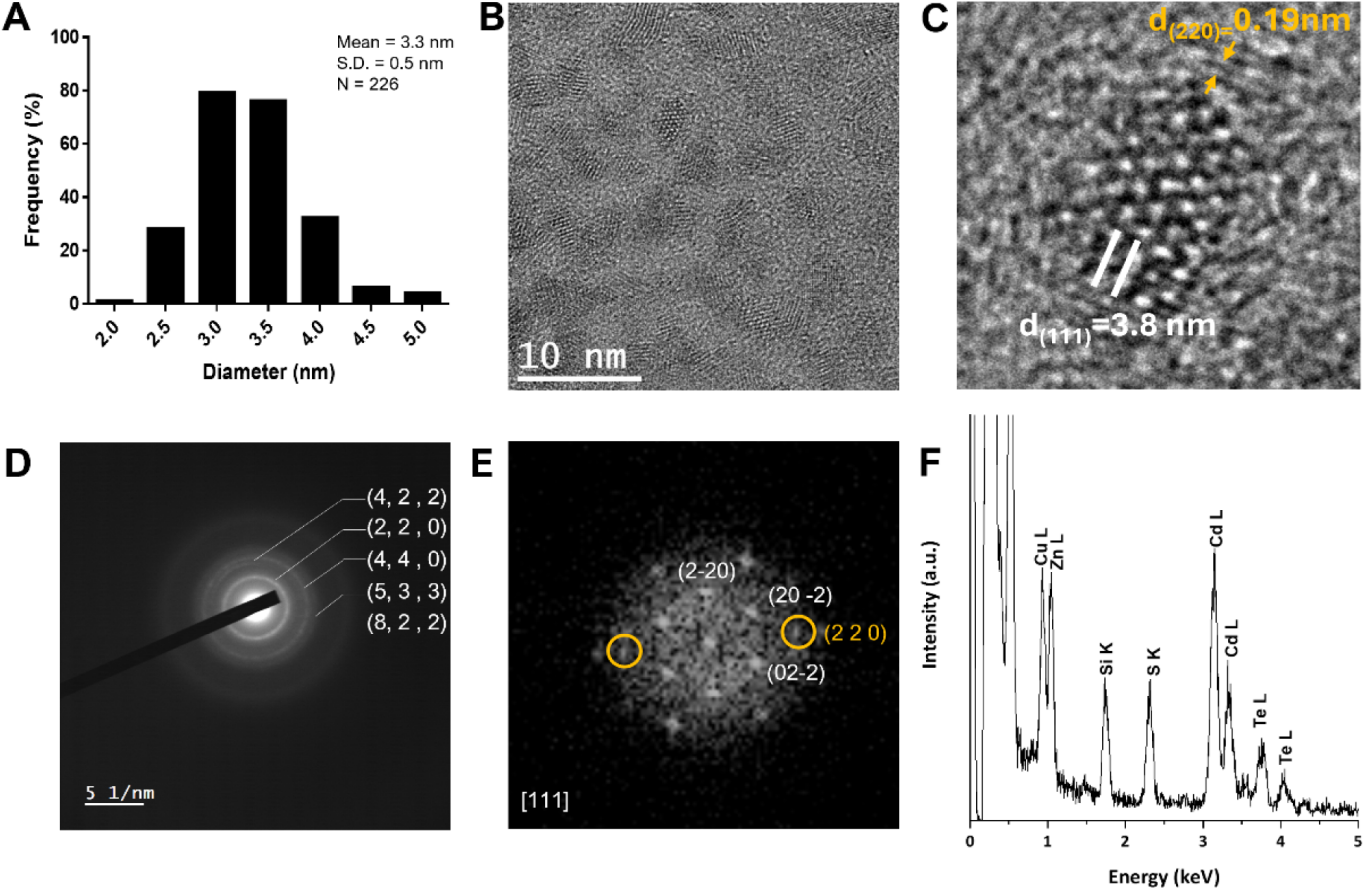
(A) Particle size distribution. (B) HRTEM image of ZnS CdTe QDs (scale bar: 10 nm). (C) HRTEM image of a single particle. (D) SAED pattern. (E) FFT of the particle in C. (F) EDX spectrum of ZnS CdTe (the Cu signal is from the TEM grid).

#### 3.1.2 Influence of His_6_-Tag-DNA Concentration

A 19 nt DNA strand (NBP770, see Table 1 for sequences) was attached to the Zn-rich QD surface via metal-affinity-mediated self-assembly of a His_6_-containing peptide that was linked to the 5′ end of the DNA. To study how the concentration of His_6_-peptide-DNA influenced the response of the nanosensor toward PCA3-DNA, we tested concentrations of DNA from 0 to 12.0 nmol L^–1^ while keeping the concentrations of QDs and Tb-DNA constant at 3.33 and 6.67 nmol L^–1^, respectively, during the preparation of the nanosensor. The signals obtained from TR PL detection from both *SPARK* and *KRYPTOR* plate readers were analyzed, and the relative intensities (eq 7) were calculated (Figure 4A,B). Increasing His_6_-peptide-DNA concentrations resulted in increasing Tb PL quenching (decreasing Tb PL intensities) until reaching saturation at approximately 8–10.0 nmol L^–1^. Considering the QD concentration of 3.33 nmol L^–1^, a maximum of three DNA strands could be attached per QD. This DNA concentration (maximum DNA attachment per QD) was used for the following assay experiments.

**Figure 4:**
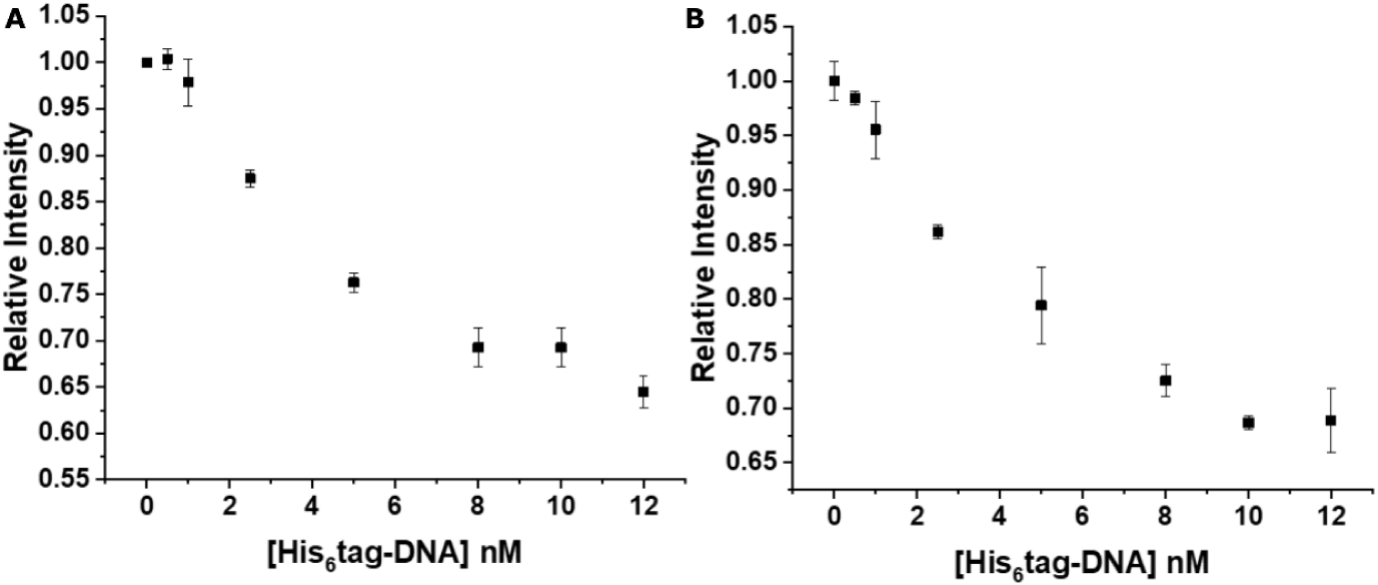
Tb PL intensity quenching as a function of His_6_-DNA concentration using a constant QD concentration of 3.33 nmol L^–1^ measured on *SPARK* (A) and *KRYPTOR* (B) plate readers. Error bars represent standard deviations (*n* = 3).

The emission spectra of the Tb donor at increasing His_6_-DNA concentrations, illustrating the photoluminescence quenching process, are provided in Figure S1.

#### 3.1.3 Influence of Tb-DNA Concentration

As the counterpart of the QD acceptor, the concentration of the Tb donor is also important for FRET assay performance. Thus, we investigated the Tb PL intensities at a constant QD-DNA concentration of 3.33 nmol L^–1^ (corresponding to 10.0 nmol L^–1^ of His_6_-peptide-DNA) for varying concentrations of Tb-DNA. As expected, both the control experiment of Tb-DNA alone (red data points in Figure 5A) and Tb-DNA in the presence of QD-DNA (black data points in Figure 5A) led to increasing Tb PL intensities with increasing Tb-DNA concentrations. However, due to Tb PL quenching via the QD acceptors, the overall PL was significantly lower when the Tb-QD FRET pair was assembled via QD-DNA/Tb-DNA hybridization. Considering a spherical QD with a radius of ca. 1.7 nm (3.3 nm diameter, vide supra), a Tb-to-QD-surface distance of ca. 4.7 nm, as previously determined with a similar His_6_-peptide-DNA assembly to a QD and Tb-DNA hybridization,^34^ and a Förster distance of *R*_0_ = 6.6 nm (vide supra), the Tb-to-QD-center distance of around 6.4 nm would result in a FRET efficiency of approximately *E*_FRET_ = 55% (eq 6). The Tb-DNA concentration-dependent Tb PL quenching results (Figure 5B) confirm that there is significant (around 35%) but not complete quenching. The slightly lower FRET efficiencies compared to the estimated value of 55% could have resulted from incomplete hybridization and errors in QD size/shape and peptide-DNA length estimations. Most importantly, significant FRET was experimentally confirmed, which was the most relevant result for demonstrating the feasibility of a FRET assay. Thus, for all assays, we selected concentrations of 3.33 nmol L^–1^ QD-DNA (corresponding to 10 nmol L^–1^ His_6_-peptide-DNA) and 6.67 nmol L^–1^ Tb-DNA.

**Figure 5:**
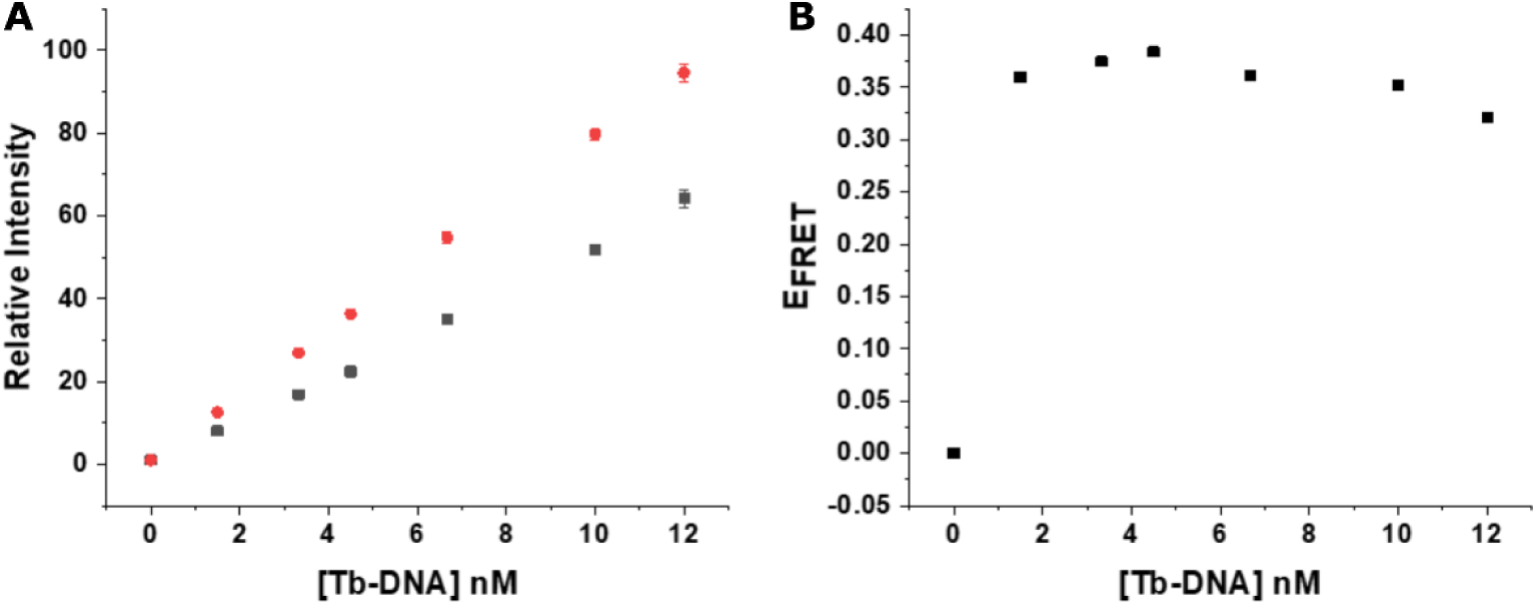
(A) Assay response curves of the probe to the assessment of the influence of the Tb-DNA concentration. Tb PL intensities were recorded at 494 nm, and the relative intensities were calculated as described in eq 7 in the “Materials and Methods” section. The probe, composed of QDs, His_6_-tag-DNA, and Tb-DNA, is represented by the gray squares, and the red circles are related to the control curve (only Tb-DNA). (B) FRET efficiency as a function of Tb-DNA concentration, calculated by eq 6.

### 3.2 Assessment of the Sensing Scheme

#### 3.2.1 Evaluation of PCA3 Response

All photoluminescence (PL) measurements in this study were performed using time-resolved (TR) detection settings on both *SPARK* and *KRYPTOR* plate readers. The use of TR-FRET, enabled by the long-lived emission of terbium donors, allowed for time-gated signal acquisition that effectively suppressed background autofluorescence from biological matrices. This significantly improved the signal-to-noise ratio and the assay sensitivity. The TR detection parameters are detailed in the “Instrumentation” section, including an excitation wavelength of 337 nm, a lag time of 100 μs, and an integration time of 2000 μs for the *SPARK* reader, as well as a 0.1–0.9 ms detection window following pulsed excitation for the *KRYPTOR* system. All calibration curves and LOD values reported are based on TR PL measurements.

In this work, we employed a synthetic DNA analogue of the PCA3 transcript as a stable RNase-resistant model to optimize the probe design, hybridization conditions, and TR-FRET signal parameters. Working first with DNA allowed us to avoid the additional complexity and variability introduced by the RNA secondary structure, enzymatic degradation, and extraction procedures from biological samples. Nevertheless, the hybridization chemistry, probe sequences, and detection workflow are directly applicable to the native lncRNA target.

Using the optimized DNA-probe conditions, we studied the assay performance for quantifying the 30 nt PCA3-DNA, i.e., the PCA3-DNA concentration-dependent displacement of Tb-DNA from the QD-DNA and concomitant Tb PL recovery (Figure 1). First, to determine the assay’s sensitivity (TR Tb PL intensity change as a function of PCA3 concentration) and limit of detection (LOD), target concentrations ranging from 0 to 5.00 nmol L^–1^ PCA3 were measured in 150 μL wells on both the *SPARK* and *KRYPTOR* plate readers. The relative TR Tb PL intensities (eq 7) were recorded as a function of PCA3 concentration to plot assay calibration curves (Figure 6), following the procedure described in the “Detection of Target PCA3” section. In this procedure, 100 μL of the probe solution (containing ZnS QDs at 3.33 nmol L^–1^, Tb-DNA at 6.67 nmol L^–1^, and His_6_-DNA at 10.0 nmol L^–1^ in HB) was combined with 50 μL of target to yield a total volume of 150 μL for measurement. Within the linear concentration range from ca. 0.10 to 5.00 nmol L^–1^, the sensitivities were 0.091 ± 0.002 nmol L^–1^ (*SPARK*) and 0.073 ± 0.007 nmol L^–1^ (*KRYPTOR*), whereas the LODs were 1.52 ± 0.36 nmol L^–1^ (*SPARK*) and 1.57 ± 0.40 nmol L^–1^ (*KRYPTOR*).

**Figure 6:**
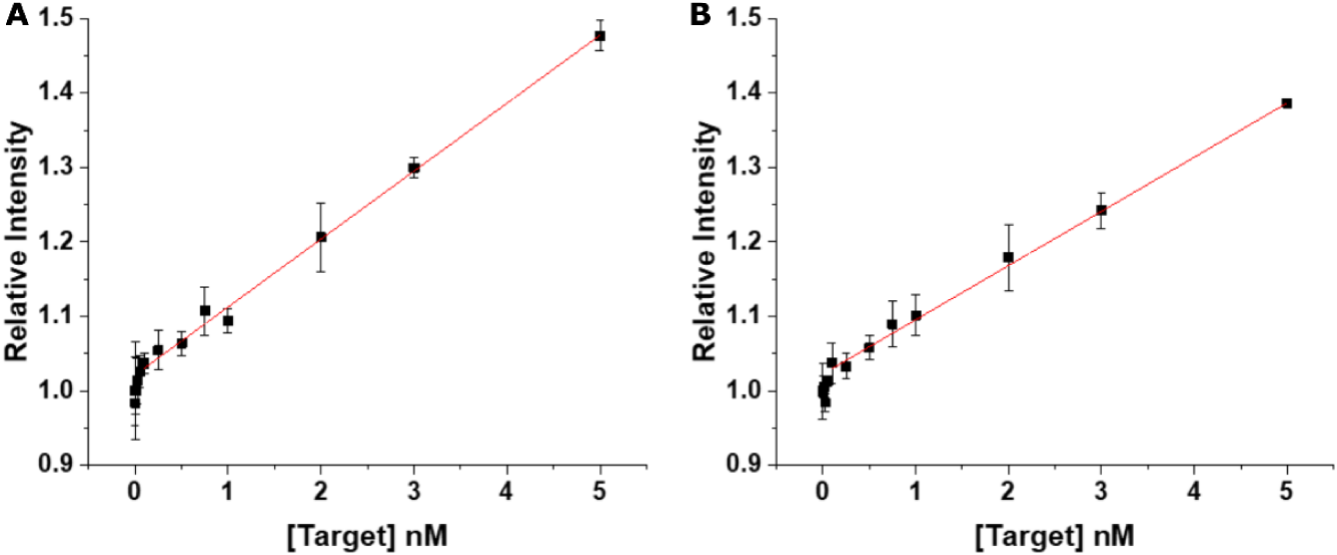
Target displacement FRET assay calibration curves obtained from (A) *SPARK* (Tecan): linearity = 0.10–5.00 nmol L^–1^, *R* ^2^ = 0.996, slope = 0.091 ± 0.002 nmol L^–1^ and (B) *KRYPTOR*: linearity = 0.10–5.00 nmol L^–1^, *R* ^2^ = 0.999, slope = 0.073 ± 0.007 nmol L^–1^. Error bars represent standard deviations *n* = 3, except the blank *n* = 10.

Using different probe concentrations can result in different slopes and dynamic ranges of the assay calibration curve. As shown in Figure S2 (and detailed in Table S1), Code 1 exhibits the lowest slope and widest range, Code 4 the steepest slope and narrowest range, while Code 2 and Code 3 occupy intermediate sensitivity–range profiles (Code 2 offering the most balanced performance). Therefore, we compared the assays at the previously determined probe concentrations of 3.33 nmol L^–1^ QD-DNA (corresponding to 10.0 nmol L^–1^ His_6_-DNA) and 6.67 nmol L^–1^ Tb-DNA with 2-fold (hereby designated as Code 2), 4-fold (Code 3), and 8-fold (Code 4) diluted probe concentrations at PCA3-DNA concentrations between 0 and 10.0 nmol L^–1^ (Figure S2 and Table S1).

Next, by testing a larger number of distinct PCA3-DNA concentrations between 0 and 10.0 nmol L^–1^, the assay employing a 2-fold diluted probe concentration (Figure 7) provided the best compromise between a relatively broad dynamic concentration range up to 1.75 nmol L^–1^, high sensitivity (0.158 ± 0.003 nmol L^–1^ for *KRYPTOR* and 0.192 ± 0.008 nmol L^–1^ for *SPARK*), and low LOD (0.32 ± 0.08 nmol L^–1^ for *KRYPTOR* and 0.65 ± 0.16 nmol L^–1^ for *SPARK*). Points near or below the LOD were included in the plot for completeness but were not considered to be part of the quantifiable range.

**Figure 7:**
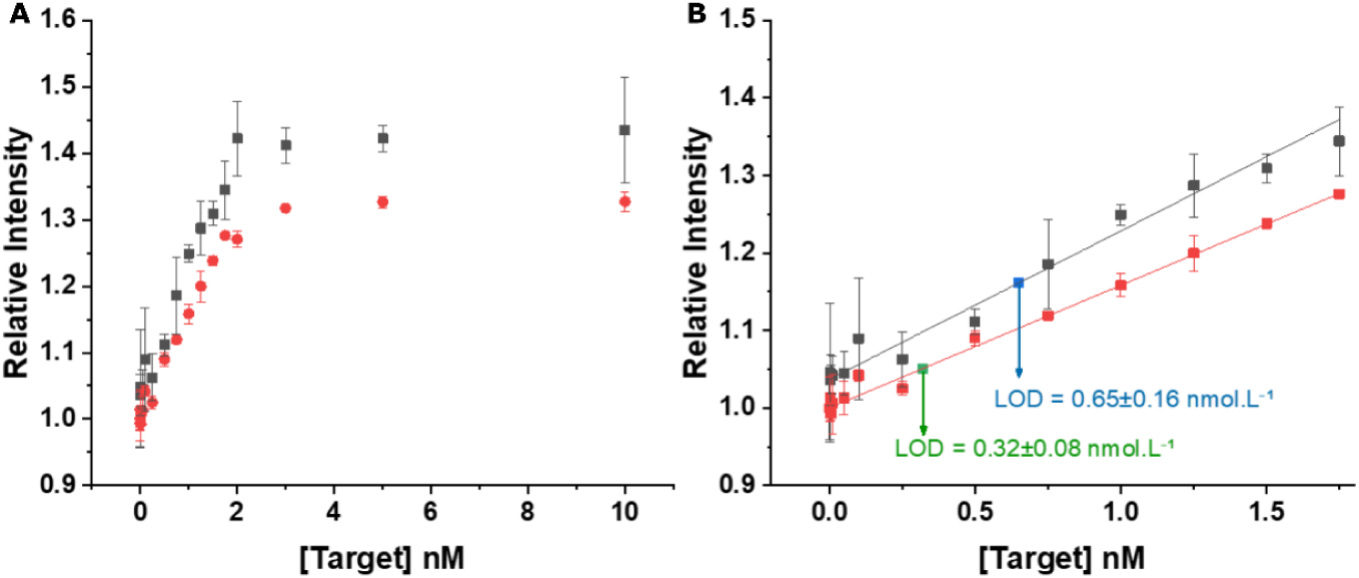
Calibration curve of the PCA3 FRET displacement assay showing the full concentration range on the left (A) and a reduced range with LOD determination on the right (B). Measurements were performed using *SPARK* (black squares) and *KRYPTOR* (red dots). Error bars indicate standard deviations, with *n* = 3 for all concentrations and *n* = 10 for the blank.

To contextualize the analytical performance of the proposed TR-FRET nanoassay, a comparative summary of key figures of merit for previously reported PCA3 detection methods is presented in Table 2.

**Table 2:** Comparative Analysis of PCA3 Detection Methods^a^

Detection method	Detection Principle/Platform	LOD	Linear Range	Target/Sample type	Validated with Real Sample?	Reference
TR-FRET (*SPARK* plate reader)	Time-resolved FRET (Tb-to-QD) DNA-displacement	0.65 nmol L^–1^	up to 1.75 nmol L^–1^	Synthetic PCA3-DNA/Buffer, 50% FBS	No	This work
TR-FRET (*KRYPTOR* clinical reader)	Time-resolved FRET (Tb-to-QD) DNA-displacement	0.32 nmol L^–1^	up to 1.75 nmol L^–1^	Synthetic PCA3-DNA/Buffer, 50% FBS	No	This work
SERS-based competitive lateral flow assay	Competitive hybridization between target and reporter DNA on test line, monitored via SERS using AuNP-based nanotags	3 fmol L–1	0.01–50 000 pmol L^–1^	Synthetic PCA3-DNA/buffer and diluted human serum	Yes	Fu et al. (2019)^11^
SERS-based DNA assay	Sandwich-type hybridization between ASO735-HGN SERS tags and ASO683-magnetic beads for PCA3-DNA, enabling magnetic separation and Raman readout	2.7 fmol L^–1^	1 fmol L^–1^ to 10 pmol L^–1^	Synthetic PCA3-DNA/distilled water	No	Yu et al. (2017)^12^
EIS + CV + UV–vis + ML	LbL genosensor (CS/AuNPs) with EIS, CV, UV–vis, and ML-assisted SEM for PCA3 hybridization detection	EIS: 83 pmol L^–1^; CV: 2000 pmol L^–1^; UV: 900 pmol L^–1^	Not stated	Synthetic PCA3-DNA/Buffer	No	Rodrigues et al. (2021)^15^
Colorimetric and spectrophotometric	Gold NPs + thiolated PCA3 products of RT-PCR	31.25 ng/reaction	Not stated	Urine PCA3-RNA/Urine	Yes	Htoo et al. (2019)^10^
Electrochemical and Electric Impedance Spectroscopy	Label-free detection using LbL biosensor with chitosan/MWCNTs functionalized with ssDNA probe for PCA3; hybridization alters interfacial impedance on interdigitated gold electrodes	0.128 nmol L^–1^ (EIS); 1.42 nmol L^–1^ (IS)	Evaluated over 10^–6^–10^–16^ mol/L; regression parameters not reported	Synthetic PCA3-DNA/total RNA from prostate cancer cell lines (LNCaP, PC3) and HeLa (cervical cancer)	Yes	Soares et al. (2019)^13^
Electrochemical (DPV)	Aptamer-based DPV using methylene blue-modified DNA probes immobilized on AuNP-modified gold electrode; hybridization reduces current signal	0.1 pmol L^–1^	0.1 pM–10 nmol L^–1^	Synthetic PCA3-RNA/PBS and synthetic urine	Yes (artificial urine)	Takita et al. (2023)^14^
Fluorescence imaging	EDRE-based DNA circuit with FAM-BHQ1 probes on Ti_3_C_2_–TAT MXenes for PCA3-triggered strand displacement and signal amplification	2.6 pmol L^–1^	0–0.2 nmol L^–1^	Synthetic PCA3-DNA/Buffer and LNCaP, PC3, RWPE-1, and A549 cells	Yes	Wang et al. (2019)^16^
Fluorescence	FRET-based quenching between upconversion nanoparticles (NaYF_4_:Yb,Er) and graphene oxide, disrupted by PCA3 hybridization	500 fmol L^–1^	Evaluated over 200 fmol L^–1^–5 nmol L^–1^; regression parameters not reported	Synthetic PCA3-DNA/Buffer, blood plasma, cell lysate	Yes	Vilela et al. (2017)^17^
Metal-Enhanced Fluorescence (MEF)	PCA3-induced hybridization assembles AuNR/Ag_2_S QD satellite nanostructures, enhancing fluorescence via plasmonic MEF effect	1.42 pmol L^–1^	5–500 pmol L^–1^	Synthetic PCA3-DNA/Buffer, human serum and cell lysates	Yes	Jia et al. (2021)^18^

a TR-FRET: Time-Resolved Förster Resonance Energy Transmission; FBS: Fetal Bovine Serum; EIS: Electrochemical Impedance Spectroscopy; CV: Cyclic Voltammetry; UV–vis: Ultraviolet-Visible Spectroscopy; LbL: Layer by Layer; CS/AuNPs: Chondroitin Sulfate/Gold Nanoparticles; ML-assisted SEM: Machine Learning + Scanning Electron Microscopy analysis; RT-PCR: Reverse Transcription Polymerase Chain Reaction; SERS: Surface-Enhanced Raman Scattering; ASO735: Antisense Oligonucleotide 735; HGN: Hollow Gold Nanosphere; ASO683: Antisense Oligonucleotide 683; EIS: Electrochemical Impedance Spectroscopy; IS: Electric Impedance Spectroscopy; DPV: Differential Pulse Voltammetry; EDRE: Entropy-Driven RNA Explore.

#### 3.2.2 Nonspecific Binding and Target Specificity

The selectivity of the proposed sensing methodology was evaluated using two different DNA target sequences (NBP353 and NBP767, cf. Table 1) that were not fully complementary to the probe Tb-DNA. The obtained relative intensities were calculated using eq 7, and the assays were compared to PCA3-DNA (Figure 8). A target-concentration-dependent PL intensity increase was only observed for PCA3-DNA, whereas the PL intensities remained at background levels for all concentrations of both noncomplementary targets, which confirmed the PCA3 specificity of the assay.

**Figure 8:**
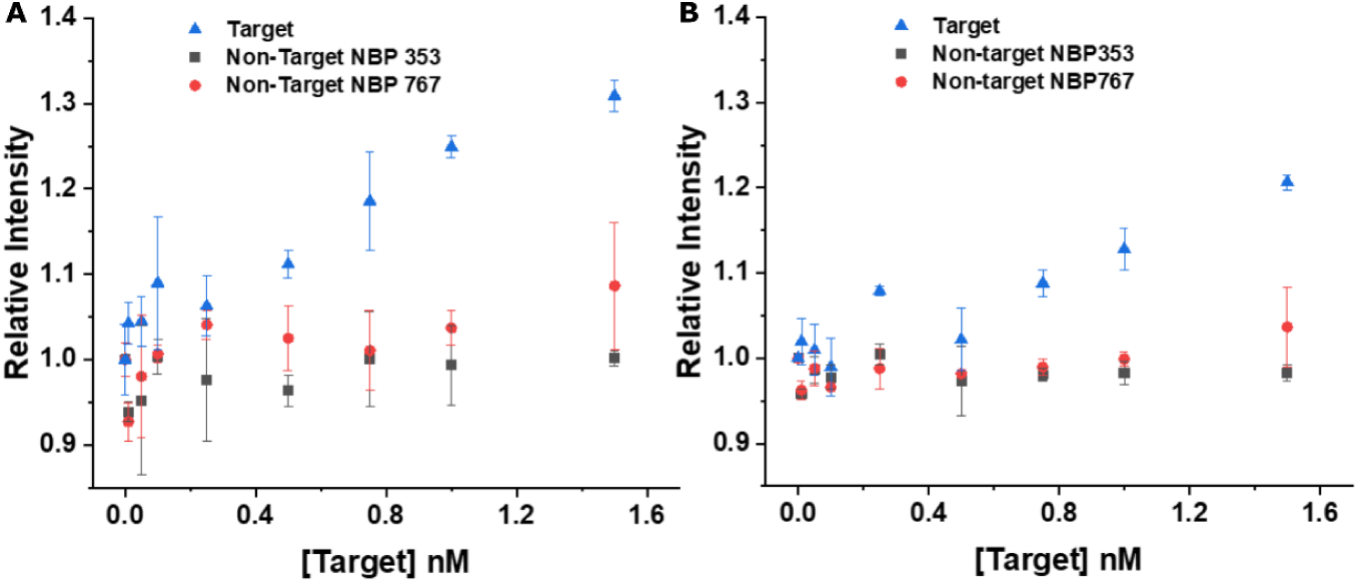
Evaluation of PCA3 assay specificity using PCA3-DNA (blue triangles) and noncomplementary targets (NBP353, black squared; NBP767, red dots). The tested concentrations were 0, 0.01, 0.05, 0.1, 0.25, 0.5, 0.75, 1, and 1.5 nmol L^–1^, and the assays were performed on *SPARK* (A) and *KRYPTOR* (B). Error bars represent standard deviations (*n* = 3).

#### 3.2.3 PCA3 Detection in Fetal Bovine Serum

Another important proof-of-concept toward real-life applicability is the evaluation of the assay performance under more complex biological conditions. Therefore, we evaluated the quantification of PCA3 in varying concentrations of fetal bovine serum (FBS). Given that numerous serum components may induce significant nonspecific binding, matrix effects, or interference from proteins and other components that can potentially compromise assay performance, it was essential to examine the behavior of the nanosensor in this environment. Accordingly, 5%, 10%, 20%, and 50% FBS samples were spiked with PCA3-DNA in the range of 0–10.0 nmol L^–1^ and assay calibration curves were recorded (Figure 9). As expected, the assay performance was the best (highest sensitivity) for measurements in buffer and decreased with increasing serum concentration. However, even for 50% serum content, the assay still showed a clear concentration-dependent PL intensity with a dynamic range similar to that for detection in buffer, which showed the applicability of the assay for serum-containing samples.

**Figure 9:**
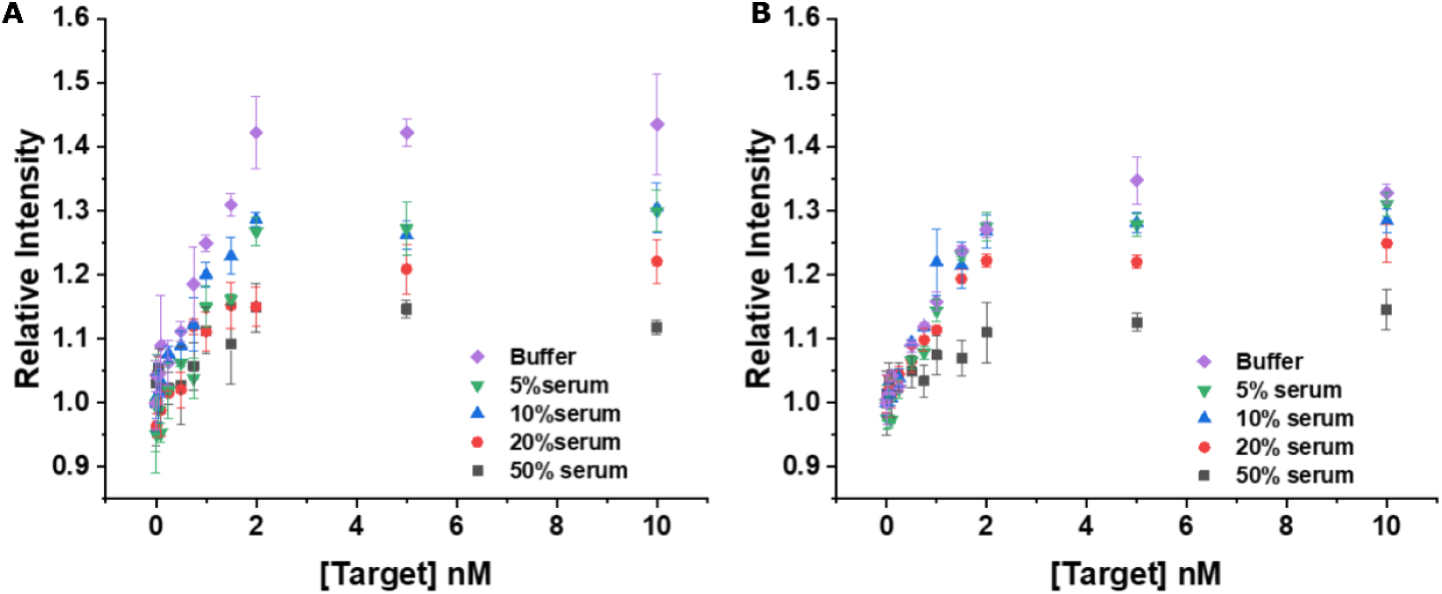
Comparison of target displacement FRET assays for PCA3-DNA in buffer (purple diamonds), 5% serum (inverted green triangles), 10% serum (blue triangles), 20% serum (red dots), and 50% serum (gray squares) measured on *SPARK* (A) and on *KRYPTOR* (B). Error bars represent standard deviations (*n* = 3).

It is important to note that all TR-FRET measurements, both in pure buffer and in serum samples, were performed in our Hybridization Buffer, which contains 0.1% (w/v) BSA. The inclusion of BSA acts as a blocking agent, reducing nonspecific adsorption of serum proteins onto the quantum dot surfaces and the microplate wells, while also partially mimicking the protein-rich environment of real biological samples. Consequently, the loss of sensitivity observed at higher FBS concentrations reflects genuine matrix interference rather than artifactual binding. Serial dilutions of serum samples (5–50% FBS) were employed to mitigate matrix effects, but in undiluted clinical specimens, a true matrix blank cannot be obtained, and residual interference inherent to complex biological matrices cannot be entirely eliminated.

In clinical practice, PCA3 expression is typically evaluated in urine samples collected after a digital rectal examination (DRE), and the result is reported as a PCA3 score, defined as the ratio of PCA3 to PSA mRNA levels. According to the FDA-cleared PROGENSA PCA3 assay,^37^ the 2025 EAU Guidelines on Prostate Cancer,^38^ and the AUA/SUO 2023 Early Detection of Prostate Cancer guidelines,^39,40^ a cutoff score of 35 is commonly used to support the indication for prostate biopsy, particularly in men with previous negative biopsies and persistently elevated PSA levels. Although our TR-FRET assay was developed using synthetic PCA3-DNA and evaluated in serum-like conditions rather than urine, the quantification range achieved (0–10.0 nmol L^–1^) and the robust, concentration-dependent signal response even in 50% serum suggest analytical performance compatible with clinically relevant transcript levels. Direct clinical comparison is not possible at this stage due to differences in matrix, biomarker format (DNA versus RNA), and normalization strategies, but these results support the potential diagnostic applicability of the assay. Future efforts toward adapting the method for long noncoding RNA detection and incorporating prostate-specific antigen (PSA) coquantification may enable a translational route toward score-based screening strategies.

## 4 Conclusions

PCA3 is an important circulating tumor marker of prostate cancer. However, most detection technologies are relatively complicated, and the development of rapid, sensitive, and specific assays has the potential to improve PCA3 testing. With this aim in mind, we developed a TR-FRET DNA-displacement assay using Tb-to-QD FRET with Lumi4-Tb complexes and small aqueous-synthesized CdTe/ZnS core–shell QDs. The mix-and-measure TR-FRET assay can address the limitations of current diagnostic methods, including the lack of standardization, overdiagnosis with subsequent overtreatment, reliance on invasive procedures, and insufficient supporting data to confirm the diagnosis.^4,6^ The rapid assay is ideally suited to complement current clinical tests based on magnetic resonance imaging, such as ConfirmMDx, Progensa PCA3, 4K Score, or Prolaris.^4^ The TR-FRET DNA-displacement nanoassay demonstrated excellent sensitivity and specificity, as evidenced by quantifying PCA3-DNA with LODs of 0.65 nmol L^–1^ when a *SPARK* benchtop plate reader was used and 0.32 nmol L^–1^ for a *KRYPTOR* clinical plate reader. Although the detection limits achieved by the developed TR-FRET assay are higher than some previously reported ultrasensitive methods, such as surface-enhanced Raman scattering (SERS)-based lateral flow assays (LFAs) capable of detecting analytes at concentrations as low as 3 fmol/L or electrochemical impedance spectroscopy (EIS) methods achieving detection limits around 83 pmol/L, the proposed TR-FRET technique offers significant practical advantages. Remarkably, TR-FRET provides faster response times due to simpler sample preparation and quicker detection workflows, making it highly suitable for rapid screening applications. Moreover, the instrumentation required for TR-FRET is generally simpler, more compact, and less expensive compared to Raman-based or electrochemical impedance spectroscopy systems, enhancing accessibility and reducing operational costs. Measurements in this study were obtained using standard laboratory multimode fluorescence plate readers (*SPARK*, Tecan; *KRYPTOR* *Compact PLUS*), highlighting the assay’s compatibility with widely available analytical equipment. The combination of these attributes, including ease of use, cost-effectiveness, portability, and rapid analysis, clearly positions TR-FRET as a valuable complementary alternative, particularly beneficial in settings where high-throughput screening and point-of-care diagnostics are prioritized over ultralow detection limits. Despite slightly reduced sensitivities, the proposed nanosensor was also functional in more challenging environments, as exemplified by samples containing up to 50% fetal bovine serum. Overall, our proof-of-concept Tb-to-QD TR-FRET assay demonstrated the potential for rapid and simple PCA3 quantification at low nanomolar concentrations using two commercially available plate reader systems, suggesting its value as a tool for prostate cancer analysis in both research and clinical laboratories.

One limitation of this research is the use of synthetic PCA3 mimic DNA instead of actual PCA3-RNA from clinical prostate cancer patients. Although synthetic mimics are a convenient first proof-of-concept to validate assay sensitivity and performance, patient-derived PCA3-RNA samples must be utilized to thoroughly confirm clinical usefulness. Future research will be focused on evaluating the TR-FRET assay constructed with clinically relevant samples, such as patients’ blood or urine, and conducting in vitro assays on cancer cell lines. The use of TR-FRET detection was a key feature of the assay, enabling efficient suppression of background autofluorescence and enhancing the signal-to-background ratio. This approach contributed to the assay’s robustness and sensitivity, even in complex biological matrices such as serum, and supports its potential for clinical translation using standard TR-FRET-capable fluorescence plate readers.

Moreover, while the current work made use of CdTe/ZnS quantum dots synthesized through an aqueous method due to their documented photophysical stability and quantum yield, we are aware of problems of potential biocompatibility and cytotoxicity due to the cadmium content. Thus, subsequent research will make use of cadmium-free analogs such as carbon dots or indium phosphide/zinc sulfide (InP/ZnS) quantum dots that offer improved biocompatibility, reduced environmental impact, and reduced toxicity profiles.

These validation steps and material modifications would significantly enhance the system’s credibility, safety, and clinical relevance, filling the gap between laboratory construction and effective diagnostic deployment.

## Supplementary Material



## Notes

Figure S1: emission spectra of the Tb donor (Lumi4-Tb-DNA) at varying His_6_-DNA concentrations; Figure S2: calibration curves of nanosensor Codes 1–4 (0–10.00 nmol L^–1^) obtained using *SPARK* and *KRYPTOR*; Table S1: the composition, calibration data, and analytical performance metrics of each code (PDF)
