# Supplementary material for: Time-Resolved Förster Resonance Energy Transfer Nanoassay Based on CdTe Quantum Dots for Sensitive Detection of Prostate Cancer Antigen 3
Source: ACS Appl Nano Mater. 2025 Jul 8;8(28):14158–69. doi: 10.1021/acsanm.5c02176 (PMC12821037; doi:10.1021/acsanm.5c02176)
Supplement: Supplementary file 1 [file an5c02176_si_001.pdf]

Supporting Information

Time-Resolved Förster Resonance Energy Transfer Nanoassay Based on CdTe Quantum Dots for Sensitive Detection of Prostate Cancer Antigen 3

Catarina S. M. Martins[1,2], Anne Nsubuga[3], Nour Fayad[3], Ruifang Su[4], Thibault Gallavardin[3], Ihsan Çaha[2], Niko Hildebrandt[5], Francis Leonard Deepak[2*], João A. V. Prior[1*]*

[1] LAQV, REQUIMTE, Laboratory of Applied Chemistry, Department of Chemical Sciences, Faculty of Pharmacy of the University of Porto, Porto, Portugal

[2] International Iberian Nanotechnology Laboratory, Braga, Portugal

[3] Laboratoire COBRA, Université de Rouen Normandie, CNRS, INSA, 76821 Rouen, France

[4] Laboratory of Biomaging and Pathologies, UMR 7021 CNRS, University of Strasbourg, Strasbourg, 67000, France

[5] McMaster University, Department of Engineering Physics, Hamilton, L8S 4L7, Canada

* Corresponding authors: João A. V. Prior, joaoavp@ff.up.pt; Francis Leonard Deepak, leonard.francis@inl.int; Niko Hildebrandt, hildebrandt@mcmaster.ca

Supporting Figures

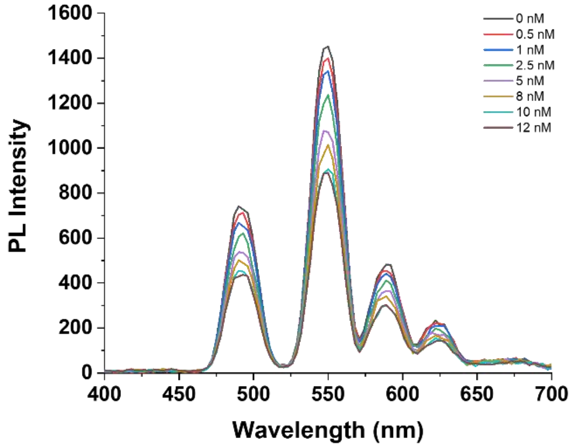

Figure S1. Emission spectra of the Tb donor (Lumi4-Tb-DNA) at varying His_6-DNA concentrations, illustrating progressive quenching of Tb photoluminescence. Spectra were recorded under identical conditions to those used for the calibration data in Figure 4A.

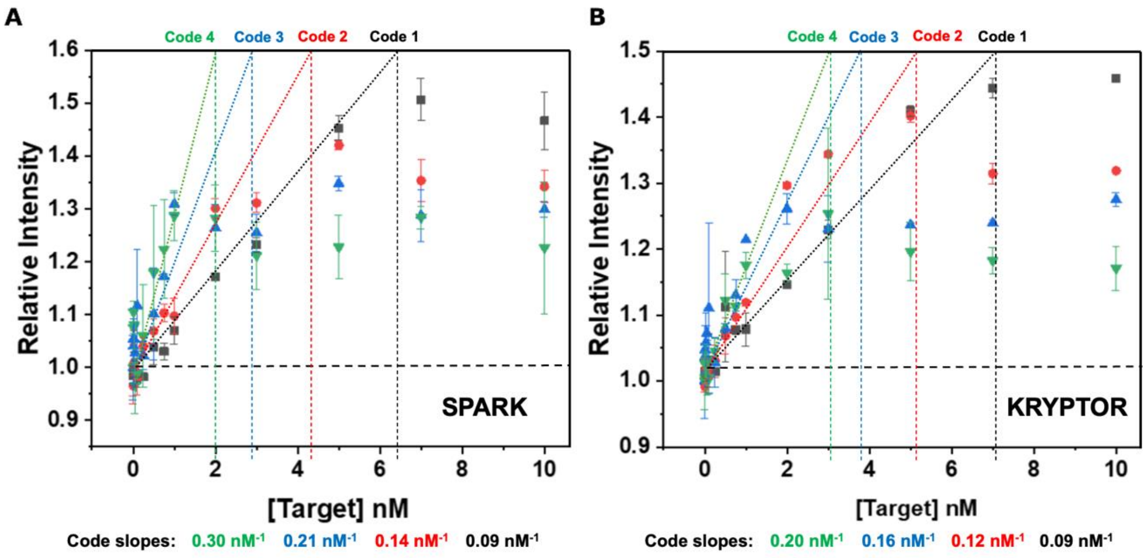

Figure S2 - Calibration curves of the different nanosensor codes from 0 – 10.00 nmol.L^{-1}. (A) Data obtained from *SPARK* and (B) data obtained from *KRYPTOR*. Code 1 (black squares), Code 2 (red dots), Code 3 (blue triangles), and Code 4 (green inverted triangles). Error bars represent standard deviations n=3, except the blank n=10.

Supporting Table

Table S1 - Compilation of composition, linearity, and figures of merit of each Code.

	Code Composition (nM)			KRYPTOR*1		SPARK*2
	**QDs**	**Tb-DNA**	**His_6-DNA**	**Linearity ($nmol.L^{-1}$)**	**Sensitivity ($nmol^{-1}.L$)**	**Sensitivity ($nmol^{-1}.L$)**
**CODE 1**	3.33	6.67	10.00	0.10 - 5.00	0.09±0.02	0.09±0.02
**CODE 2**	1.67	3.33	5.00	0.00 - 1.75	0.12±0.03	0.14±0.03
**CODE 3**	0.83	1.67	2.50	0.00 - 1.00	0.16±0.04	0.21±0.04
**CODE 4**	0.42	0.83	1.25	0.05 - 1.00	0.20±0.06	0.30±0.06

*1 *KRYPTOR compact PLUS* clinical fluorescence plate reader (Thermo Fisher Scientific)
*2 *SPARK* multimode fluorescence plate reader (Tecan)
